# Head and Neck Squamous Cell Carcinoma Vaccine: Current Landscape and Perspectives

**DOI:** 10.3390/cimb45110577

**Published:** 2023-11-16

**Authors:** Piero Giuseppe Meliante, Carla Petrella, Marco Fiore, Antonio Minni, Christian Barbato

**Affiliations:** 1 Department of Sense Organs, Sapienza University of Rome, Viale del Policlinico 155, 00161 Rome, Italy; 2 Institute of Biochemistry and Cell Biology (IBBC), National Research Council (CNR), Department of Sense Organs, Sapienza University of Rome, Viale del Policlinico 155, 00161 Rome, Italy; 3 Division of Otolaryngology-Head and Neck Surgery, Ospedale San Camillo de Lellis, ASL Rieti-Sapienza University, Viale Kennedy 1, 02100 Rieti, Italy

**Keywords:** head and neck squamous cell carcinoma (HNSCC), therapeutic cancer vaccine, Epstein–Barr virus (EBV), human papilloma virus (HPV), mRNA vaccine

## Abstract

The treatment of unresectable or metastatic Head and Neck Squamous Cell Carcinoma (HNSCC) has traditionally relied on chemotherapy or radiotherapy, yielding suboptimal outcomes. The introduction of immunotherapy has significantly improved HNSCC treatment, even if the long-term results cannot be defined as satisfactory. Its mechanism of action aims to counteract the blockade of tumor immune escape. This result can also be obtained by stimulating the immune system with vaccines. This review scope is to comprehensively gather existing evidence and summarize ongoing clinical trials focused on therapeutic vaccines for HNSCC treatment. The current landscape reveals numerous promising drugs in the early stages of experimentation, along with a multitude of trials that have been suspended or abandoned for years. Nonetheless, there are encouraging results and ongoing experiments that instill hope for potential paradigm shifts in HNSCC therapy.

## 1. Introduction

Head and neck squamous cell carcinoma (HNSCC) affects 450,000 individuals per year, accounting for an estimated 890,000 new cases, i.e., roughly 4.5% of all cancer diagnoses. The HNSCC incidence includes 380,000 cases of cancer of the oral cavity and the lip, 133,000 of the nasopharynges, 98,000 of the oropharynges, 84,000 of the hypopharynxes, 185,000 of the larynxes, and 54,000 of the salivary glands. The incidence and mortality rates of HNSCC are distributed across geographic regions and demographic traits, with a higher occurrence in men than in women and a male-to-female ratio of 2:1 [1].

In situations where surgical intervention is not viable, chemotherapy and radiation therapy are used. However, results are not optimal, particularly for recurrent or metastatic malignancies. The introduction of immunotherapy has improved those outcomes. Currently approved drugs for the treatment of HNSCC are pembrolizumab (KEYTRUDA, Merck and Co., Rahway, NJ, USA) and nivolumab (OPDIVO, Bristol Myers Squibb, New York, NY, USA), which have improved traditional chemotherapy results. Checkmate-141, Keynote-040, and Keynote-048 trials are milestones in HNSCC treatment and set a new standard of results for non-surgical therapy. However, they still are not satisfactory with long-term efficacy in 20 to 30% of patients only. The discussion of resistance mechanisms to immune checkpoint inhibitors is beyond the scope of this manuscript, but we can state that solutions need to be found to achieve better therapy performance [2,3,4].

In this scenario, the advancement of novel non-surgical therapies appears to be imperative. HNSCC are categorized as HPV-negative and HPV-positive. Tobacco consumption is the primary risk factor for the development of HPV-negative HNSCC. In addition, betel quid, areca nut, exposure to environmental pollutants, or excessive alcohol consumption is known to synergize with tobacco use to promote carcinogenesis [5,6]. While the tobacco- and alcohol-related neoplasms are decreasing, viral infection is on the rise. HPV is a common risk factor for HNSCC, being associated with oropharyngeal cancers (>70%) [7]. HPV vaccines have been approved for cervical cancer prevention in females, and their impact on HNSCC incidence has been observed [6]. However, therapeutic vaccines for HNSCC are still undergoing investigation. Their objective is to induce immunogenicity against HNSCC cells employing a range of mechanisms, including traditional approaches like cell-mediated cytotoxicity induced by antigens, as well as innovative strategies to counteract tumor immune escape mechanisms or stimulate the immune system’s cytotoxic activity against neoplastic cells [5]. Here we show an in-depth review of the limitations of current studies and future perspectives in immunotherapy for the treatment of HNSCC to provide a comprehensive overview.

## 2. Materials and Methods

**Figure 1:**
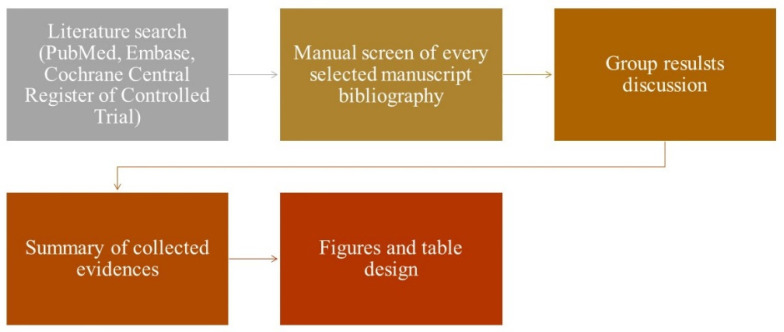
Article selection and discussion process.

A literature search was conducted across PubMed, Embase, and Cochrane Central Register of Controlled Trials databases, without any time restrictions. The search terms specifically focused on head and neck cancer vaccines, and each author independently performed the search and analysis. In addition to the database search, the authors manually screened the reference lists of retrieved articles for further relevant studies. We excluded non-English language papers. We hand-searched the ClinicalTrials.gov database for any relevant trial and checked the actual status of each of those considered. All authors discussed results with conflicts solved by our senior author, A.M. The evidence extracted from these papers was organized into coherent paragraphs. Furthermore, a thorough examination of the current studies’ limitations and future perspectives was conducted in order to provide a comprehensive overview (Figure 1). Furthermore, it is important to note that Cetuximab was approved in 2006 as the first monoclonal antibody (mAb) approved by the Food and Drug Administration (FDA), directed against the epidermal growth factor receptor (EGFR) in patients with HNSCC. Therefore, the literature timeline and considered studies utilized to perform this analysis were mainly from the period of 2016 to 2022.

### 2.1. Immunosurveillance and Immune Escape Mechanisms

The immune system plays a crucial role in the uncontrolled growth and spread of neoplastic cells, as seen via immunosurveillance [7]. It can eliminate cancer cells that are constantly produced throughout an individual’s life. During neoplastic progression, several mutations in cancer cell DNA permit the acquisition of the ability to evade the immune response through the downregulation of HLA antigens, a decrease in or loss of expression of tumor-associated antigens, and the production of immunosuppressive cytokines [8].

Tumor antigens are taken up by antigen-presenting cells (APCs), such as dendrites, and are exposed to host T lymphocytes, which in turn form effector and memory T lymphocytes. Cytotoxic T lymphocytes and natural killer cells circulate in the peripheral blood and lymphoid and non-lymphoid tissues, as well as in tumoral tissues [9]. T cells encounter tumor cells equipped with these same antigens and become activated and kill them through a cytotoxic mechanism. Cancer cell death is induced via the release of cytokines such as interferon (IFN)-γ, tumor necrosis factor (TNF)-α, granzyme, perforins, and IL-2 [10,11]. To proliferate freely, tumors may develop immune escape mechanisms via cytotoxic T-lymphocyte protein 4 (CTLA4) and programmed cell death protein 1 (PD-1). Immunotherapy drugs have been developed to inhibit these molecules and allow the immune system to act on tumors [2,3]. In addition to the molecular mechanisms of immune escape expressed on the surface of tumor cells, there are several others that involve the tumor microenvironment. These include increases in the percentage of immunosuppressive cells in the tumor matrix (myeloid-derived suppressor cells, tumor-associated macrophages, and T regulatory cells); the secretion of immunosuppressive molecules, such as transforming growth factor-β (TGF-β); and the signal transducer and stimulator of transcription (STAT)-3, as well as the formation of physical barriers and an intricate vascular network that physically prevents the penetration of immune cells into the tumor matrix [5,12].

### 2.2. Anticancer Vaccine Categories

The anticancer vaccines under study can be divided into two groups: traditional vaccines that induce a T cell-mediated immune response against specific tumor antigens, and less conventional vaccines targeting immune escape mechanisms [5,11].

Autologous vaccines, which utilize patient-specific antigens, offer a highly targeted and specific immune response. However, the complex process, including the extraction and inactivation of tumor cells, as well as the associated development costs and lack of standardization, pose challenges to their widespread use. As a result, recombinant vaccines, which are generated through the laboratory synthesis of tumor antigens, are being subjected to more extensive trials. These recombinant vaccines offer potential advantages in terms of standardization and scalability [5].

### 2.3. Anticancer Vaccine Antigens

The antigens expressed by tumors are divided into vague categories, and some antigens may belong to more than one. According to the classification of Zarour et al., those categories are oncofetal, oncoviral, overexpressed/accumulated, cancer-testis, linear-restricted, mutated, post-translationally altered, or idiotypic antigens (Table 1) [13,14,15,16]. Tumor antigens recognized by T lymphocytes can also be classified as shared antigens, tumor-associated antigens, and tumor-specific antigens, according to Coulie et al. [17].

### 2.4. Anticancer Vaccine Platforms

**Figure 2:**
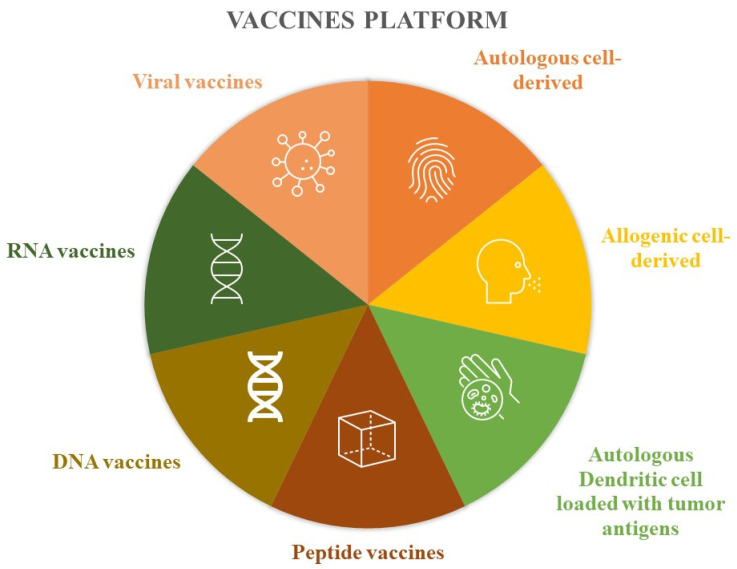
Vaccine platforms.

Devaraja et al. [5] conducted a classification of anticancer vaccine platforms, elucidating the advantages and disadvantages associated with each of them (Figure 2).

These vaccines involve extracting tumor cells from the patient, which are then inactivated in the laboratory. Subsequently, these inactivated tumor cells are combined with immunostimulant substances before being administered back to the patient. Another approach mentioned is the utilization of allogeneic tumor lines, where cells from different individuals are inactivated and used for vaccine development. Instead of using the entire tumor cell, some authors have experimented with the use of tumor antigens loaded inside the patient’s own dendritic cells, which are then reinfused to stimulate the immune response. Protein components have also been used to produce these drugs, such as peptide vaccines based on epitopes obtained through the combination of MHC class I and tumor antigens. Like proteins, nucleic acids have also been the basis of vaccines, both for DNA and RNA. Finally, viruses with low pathogenicity have also been modified to express neoplastic antigens and induce immunogenicity (Table 2) [5].

### 2.5. Virus Infection-Based Cancer Vaccines

#### 2.5.1. Epstein–Barr Virus (EBV)-Related Nasopharyngeal Carcinoma (NPC) Vaccines

Therapeutic vaccination has been extended to NPC in view of its association with EBV. The approaches developed are based on a dendritic cell-based strategy and use virus-based vaccines. Virus-induced malignancies have multiple therapeutic targets due to non-self-origin.

The Epstein–Barr virus nuclear antigen-1 (EBNA1) and the Epstein–Barr virus latent membrane proteins 1/2 (LMP1/2) are target antigens. Four trials (NCT01256853, NCT01800071, NCT01147991, NCT01094405) investigated the efficacy of EBNA1 C-terminal/LMP2 chimeric protein-expressing recombinant modified vaccinia, the Ankara vaccine (MVA) [18].

**Figure 3:**
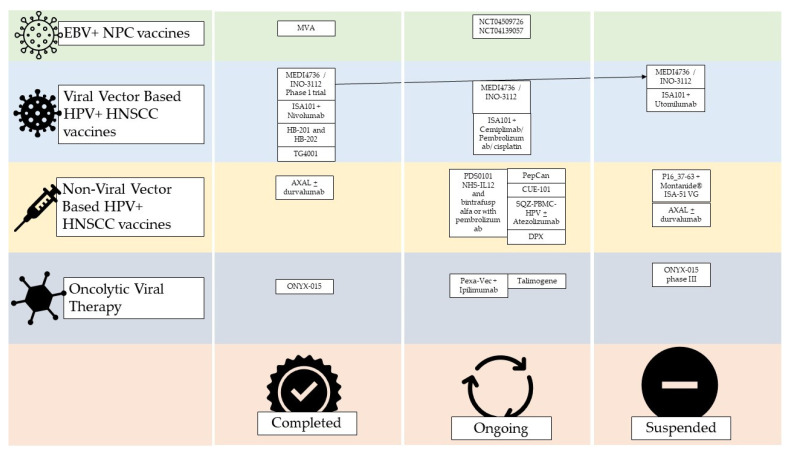
Current status of virus based HNSCC vaccine trials on humans. Unless otherwise stated, the studies considered were phase 1 or 2. Completion does not indicate the success of the therapy, but only the end of the study and the publication of its data. EBV = Epstein–Barr virus; NPC = nasopharyngeal carcinoma; HPV = human papilloma virus; HNSCC = head and neck squamous cell carcinoma; MVA = modified Ankara vaccine; DPX = DepoVax^TM^.

The study of Trabecutel (Atara Biotherapeutics) and allogenic EBV-T-cell immunotherapy was suspended after phase 1B by the sponsor and phase 2 was never conducted. The trial NCT03769467 was a multicenter, open-label, single-arm phase 1B/2 study to assess the safety and efficacy of Trabecutel in combination with pembrolizumab for the treatment of platinum-pretreated patients with recurrent/metastatic EBV+ NPC (Figure 3).

The NCT04139057 trial is recruiting patients for a phase 1 study on the administration of EBV-specific engineered T cells bearing a TCR (TCR-T) anti-PD-1. The estimated enrollment will be 18 participants affected by EBV+ HNSCC with a single-arm trial design. The estimated study completion date is 1 January 2024 (Figure 3).

TCR-Ts are the subject of a trial that is about to end (August 2023). In this single-arm study, the TCR-Ts are specific for EBV and are equipped with a cytokine-secreting system. The rationale is that cytokines, by activating both innate immunity with NK cells and adaptive immunity, promote the immune response against cancer. The study has an estimated enrollment of 20 patients pre-conditioned with chemotherapy who will be infused with EBV-specific TCR-T cells with cytokine auto-secreting element (NCT04509726) (Figure 3).

#### 2.5.2. HPV+ HNSCC Vaccines

##### Viral Vector-Based HPV+ HNSCC Vaccines

HPV+ HNSCC vaccines are different from prophylactic HPV vaccines such as Gardasil (Merck and Co., Rahway, NJ, USA) and Cervarix (GlaxoSmithKline Biologicals, Rixensart, Belgium), which target the L1 capsid protein of the virus. Infected cancer cells do not express L1, but they need the oncoproteins E6 and E7, which are induced by the virus. The therapeutic vaccines under development target those proteins from HPV-16 and -18.

For example, MEDI4736 also known as INO-3112 is a DNA-based vaccine with two components, one targeting E6 and E7 antigens from HPV-16 and -18 and another that encodes for a recombinant interleukin IL-12. The vaccine has been studied in a phase Ib/II trial involving 18 HNSCC HPV+ patients and 18 out of 21 showed antigen-specific T cell activity and persistent cellular response after 1 year. The authors concluded that INO-3112 can generate durable peripheral and tumor immune responses and hypothesized that it could be used in association with immune checkpoint inhibitors [19]. INO-3112 was studied in the suspended NCT04001413 studies and two studies in combination with durvalumab in the treatment of recurrent or metastatic HNSCC. In the first, 35 patients were enrolled, but 17 patients died during the study and 13 did not complete follow-up (NCT03162224); the second, preliminary unpublished data, and the study population was composed of any HPV+ cancer (not just head and neck) and to date, the study is indicated as ‘active’ and ‘not recruiting’ (NCT03439085) (Figure 3).

ISA101, a synthetic long-peptide HPV-16 vaccine inducing HPV-specific T cells, was studied in combination with Nivolumab in 24 patients, including 22 with oropharyngeal cancer (phase 2). The authors observed an overall response rate of 33%, with a median duration of response of 10.3 months and a median overall survival of 17.5 months, and only two grade 3 or 4 toxicity events were reported [20]. The efficacy of ISA101 in combination with Cemiplimab, Pembrolizumab, cisplatin, or Utomilumab, is ongoing (phase2), but no preliminary results have been published (NCT03669718, NCT04369937, NCT04398524). In addition, the association of ISA101 and Utolimumab (NCT03258008) was discontinued (Figure 3).

Choriomeningitis lymphocytic virus and Pichinde virus were used as two vaccines against the HPV16 E6E7 fusion protein. HB-201 and HB-202 were evaluated in the NCT04180215 trial. It is interesting to observe how intratumoral administration is being evaluated for these vaccines, alone or combined with systemic administration, as well as parenteral administration. Furthermore, the authors also experimented with the alternating administration of the two drugs, observing greater immunogenicity than with the exclusive use of one of the two (Figure 3) [21].

The vaccine TG4001 (Tipapkinogene sovacivec) is formed via an attenuated viral vector expressing the coding sequences for the E6 and E7 proteins of HPV-16 and -18, and IL-2. A phase 1B/2 study was conducted, and among nine patients enrolled, five with head and neck cancer, only three showed T-mediated peripheral immunity against E6/E7, and four showed increased CD8 infiltrate and/or T-reg/CD8 ratio in the neoplastic tissue (Figure 3) [22].

##### Non-Viral Vector-Based HPV+ HNSCC Vaccines

SQZ-PBMC-HPV (SQZ Biotechnologies, Watertown, MA, USA) is a vaccine produced using a proprietary technology called cell squeeze technology, which acts on circulating mononuclear cells. Phase 1 trial NCT04084951 evaluated its safety and efficacy in monotherapy and association with atezolizumab (Tecentriq) or any other ICI, in patients with locally advanced, recurrent, or metastatic HPV+ cancers, including HNSCC. Preliminary results showed good tolerability and immune response, even though the HNSCC population was only 3 out of the total 12 (Figure 3) [23].

PDS0101 is a liposomal-based vaccine against HPV16 E6 and E7 proteins that also contain R-DOTAP, a lipid under evaluation for anti-HPV+ and HNSCC activity. This vaccine is under study in combination with NHS-IL12 and bintrafusp alfa or with pembrolizumab. NHS-IL12 is an immunocytokine that results in IL-12, and bintrafusp alfa is a molecule obtained by combining a human IgG1 against PD-L1 and the extracellular domain of the TGF-β receptor type II, and the result is an action against TGFβRII. Their associations with PDS0101 are under evaluation in the NCT04287868 trial, whose recruitment is expected to end on 1 January 2024, and no preliminary results have been published yet. The case of the association with pembrolizumab in the trials NCT04260126 (VERSATILE002) and NCT05232851 is different, as preliminary results have been disclosed. The phase II study VERSATILE002 has a population of patients affected by recurrent or metastatic HNSCC, who are positive for both HPV 16 and PD-L1. The phase 1/2 trial NCT05232851, however, has a population of patients with locally advanced squamous cell carcinoma of the oropharynx. PDS0101 and pembrolizumab were well tolerated with no significant toxicity in the enrolled population [24]. Their combination showed significant anti-tumor activity and the FDA granted the Fast Track designation to this association for use in recurrent or metastatic HPV16+ and HNSCC (Figure 3) [25].

AXDS 11-001, also known as Axalimogene Filolisbac or AXAL (Advaxis Inc., Princeton, NJ, USA), is based on the bacterium Listeria monocytogenes listeriolysin O, modified to secrete the HPV-E7 tumor antigen as a fusion protein called LLO-E7 [26]. The NCT02002182 trial enrolled 15 patients divided into two groups, in one of which the vaccine was administered before transoral robotic surgery in the treatment of squamous cell carcinoma of the larynx, while the other group was directly subjected to surgery to evaluate the immune response induced by the vaccine. Only nine patients completed the study, five in the experimental group and four in the control group. ADXS 11-001 showed increased systemic immune response and CD4+ and CD8+ T cell infiltration. At the same time, the vaccinated subjects had an incidence of adverse events of 55.5% compared to 16.7% in the control group [26]. The suspicion of adverse events associated with this type of drug seems to have been increased by two further trials, NCT02291055 and NCT01598792. The first involved a combination of AXAL and durvalumab and was put on hold due to the death of a patient. The second, concerning HPV16+ oropharyngeal carcinoma, had only two patients enrolled and was suspended because one experienced dose-limiting toxicity (Figure 3) [27].

The DepoVax^TM^ (DPX)-E7 (IMV Inc., Dartmouth, NS, Canada) is an HPV-16 E711-19 nanomer that demonstrated antigen-induced effective anti-cancer immunity in mice models [28]. It is under study in a phase 1b/2 trial for HPV+ head and neck, cervical, or anal cancer (positive for HLA-A*02) in the clinical trial NCT02865135. Eleven patients have been enrolled and are currently under follow-up, the estimated completion time is December 2023. No preliminary data have been published yet (Figure 3).

Another peptide-based vaccine which, to date, has only been tested in animal models is based on the intratumoral injection of the E7 long peptide. This practice effectively controlled buccal TC-1 cancers in mice models and enhanced E7-specific CD8 intratumoral and circulating cells. The immune reaction induced in those animals was not dependent on CD4+ T cells (Figure 3) [29].

A novel technique for vaccine development is Immuno-STAT. These are fusion proteins built to deliver cytokines to achieve specific CD8+ T cell activation. CUE-101 is the first Immuno-STAT-based vaccine in a clinical trial, and it is composed of a human leukocyte antigen (HLA) complex (HLA-A*0201), an HPV16 E7 protein-derived peptide epitope, and four reduced-affinity IL-2. The vaccine was designed to induce HPV-16-specific CD8+ T cell activation. The first phase 1 trial is going to be completed in December 2023 with a population of 85 patients with HPV16+ recurrent/metastatic HNSCC as a monotherapy treatment in the second line or combination therapy with Pembrolizumab in the first line (NCT03978689). Partial data from this trial suggest the selective expansion of HPV-16-specific CD8+ T cells and good tolerability. Data about efficacy are still limited with 1 of 14 patients exhibiting a partial response and 6 of 14 patients exhibiting stable disease for more than 12 weeks in the CUE-101 arm, and 2 of 7 patients exhibiting a partial response and 2 of 7 patients exhibiting stable disease in the combination arm (Figure 3) [30].

The combination of HPV-16 E6 peptides and Candida skin test reagent as a novel adjuvant was used to create the PepCan vaccine [31,32]. For the positive treatment of cervical cancer [33], this vaccine is being studied for HNSCC in the NCT03821272. The investigators are giving PepCan or placebo to patients affected by HNSCC who achieved remission for a period of 2 years.

The University of Southampton, in collaboration with BioNTech SE, is carrying out the HPV Anti-CD40 RNA vaccinE (HARE-40) phase 1/2 vaccine dose escalation study, in which they are analyzing the BNT113 (an anti-CD40 RNA vaccine from BioNTech SE, Mainz, Germany) as a monotherapy. The trial has two arms, the 1A is an intrapatient dose escalation in patients with previously treated for HPV16+ head and neck cancer using two dose cohorts to establish a safe, tolerable, and recommended dose of the HPV vaccine. Arm 1B will perform a dose escalation in patients with advanced HPV16+ cancer (head and neck, anogenital, penile, cervical, and other) using a single cohort to establish a safe, tolerable, and recommended dose of HPV vaccine. The estimate study completion date is 30 April 2025. However, recruitment is still suspended due to the COVID-19 pandemic (NCT03418480).

Classically, HPV E6 and E7 have been used as target antigens in HPV+ HNSCC development. Some studies observed the hyperexpression of p16^INK4a^ and studied it as a target. The NCT02526316 VICORYX-2 trial evaluated the combination of P16_37-63 peptide combined with Montanide^®^ ISA-51 once a week for 4 weeks. The trial included patients with HPV+ cancer with a diffuse expression of p16^INK4a^ (not only head and neck but also anogenital cancers). The investigators enrolled 11 patients, and the study was completed in May 2017. No data have been published yet. A phase 1/2a trial studied the combination of the peptide P16_37-63 and Montanide^®^ ISA-51 VG regarding safety and efficacy. A total of 26 patients with HPV+ SCC (anogenital and head and neck) were enrolled and after an initial safety assessment of 10 of these, the researchers studied the efficacy of the medication. A total of 20 patients received at least four doses of the vaccine and were evaluated for immune response. CD4+ cells were induced in 11 out of 20 patients, CD8+ in 4 out of 20, and antibodies in 14 out of 20. None of the patients healed, but 10 of them had stable disease, of whom 3 were stable for the whole duration of the follow-up (NCT01462838). The trial was prematurely discontinued due to premature death or progressive disease in most of the patients (Figure 3) [34].

### 2.6. Oncolytic Viral Therapy

The use of viruses as weapons to kill cancer cells was pioneered over 20 years ago. ONYX-015 first entered clinical trials in 1996; it is an adenovirus with a deletion of the E1B gene engineered to selectively lyse p53-deficient neoplastic cells and not attack healthy cells. The drug has been tested using intratumor administration. Post-treatment biopsies showed the presence and/or replication of the virus in 7 of 11 patients in the tumor but not in the immediately adjacent tissues. A total of 21% of patients showed tumor regression with a volume greater than 50% and no alterations of the surrounding tissues [35,36]. Intratumoral ONYX-015 has also been studied in combination with the systemic administration of cisplatin and 5-fluorouracil. The rationale behind Khuri et al. attempting this approach was that while ONYX-015 demonstrated efficacy in HNSCC, the disease rapidly relapsed. The scholars observed a response in all patients treated with the combination, while the group treated only with traditional chemotherapy underwent progression. Again, the intratumor replication of the virus was confirmed by biopsies [37]. In 2001, the use of ONYX-015 was also tested intravenously in patients with metastatic solid tumors. The authors observed an increase in neutralizing antibodies and several inflammatory cytokines. But, in this study, only two of the patients had HNSCC [38]. Given the promising results of the phase 2 studies regarding ONYX-015, a phase 3 study has been reported to have taken place more than 20 years ago, but no data are available [39,40,41].

Pexa-Vec is an oncolytic virus vaccine derived from the Wyeth-strain that has been genetically modified to express the huma GM-CSF. The mechanism of action includes the activation of dendritic cells and the enhancement of the tumor immune infiltrating cells. The association of Ipilimumab with the treatment of locally advanced, recurrent, or metastatic solid cancers, including HNSCC, is under evaluation in the NCT02977156 trial.

Talimogene is derived from the herpes virus carrying GM-CSF, and its association with pembrolizumab demonstrated a tolerable safety profile, but the efficacy was similar to that of pembrolizumab monotherapy in historical HNSCC trials (Figure 3) [42].

H101 is another oncolytic adenovirus-based vaccine like ONYX-015. Its intratumorally administration associated with systemic chemotherapy has been compared with chemotherapy alone. The combination arm showed a higher response rate (79% vs. 39.6%, *p* < 0.001). In 2005, the Chinese government approved the H101 vaccine in combination with cisplatin-based chemotherapy for the treatment of nasopharyngeal carcinoma (Figure 3).

### 2.7. Cancer Testis Antingen-Based Vaccines

**Figure 4:**
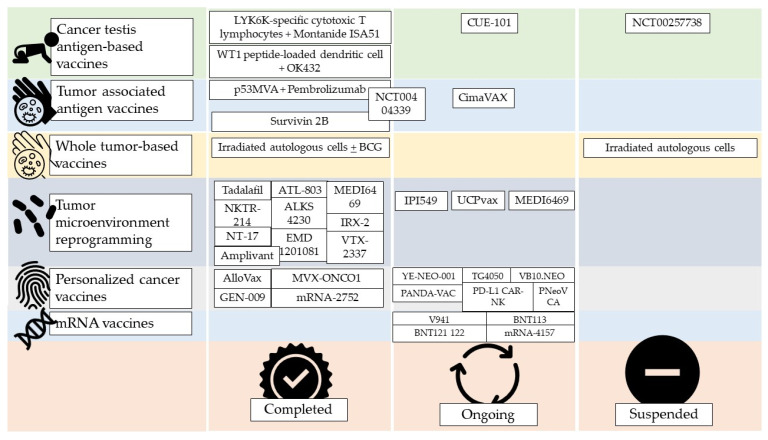
Current status of non-virus-based HNSCC vaccine trials on humans. Unless otherwise stated, the studies considered were phase 1 or 2. Completion does not indicate the success of the therapy but only the end of the study and the publication of its data. MVA = modified Ankara vaccine.

The most frequently over-expressed cancer testis antigens in HNSCC are from the MAGE group [43]. A pilot study using Trojan vaccines demonstrated acceptable toxicity and systemic immune responses against HLA-II-restricted epitopes in five MAGE-A3/HPV 16+ patients with recurrent or metastatic (R/M) HNSCC. Montanide ISA 51 and GM-CSF were used as adjuvants to facilitate dendritic cell migration to the vaccination site and enhance antigen presentation [44]. A phase 1 trial (NCT00257738), involving additional cases of progressive recurrent or metastatic HNSCC (HLA A2+), confirmed the feasibility and safety of these vaccines. Unfortunately, the trial, originally intended to enroll 90 cases, prematurely closed due to poor accrual after enrolling only 17 patients (Figure 4). Any immunized patients in both studies demonstrated partial or complete clinical response. The efficacy of a dual-antigenic peptide vaccine comprising MAGED4B and four-jointed box 1 (FJX1) was studied, evidencing strong immunogenic responses with the peptide combination compared to individual use. These have only been studied in vitro or in mouse models (Figure 4) [45,46].

Another peptide that has been studied in vaccine development is LY6K. It is overexpressed in HNSCC and undetectably low in normal cells. A vaccine based on LYK6K-specific cytotoxic T lymphocytes has been studied in 37 patients affected by recurrent or metastatic HNSCC along with Montanide ISA51 as an adjuvant. This therapy was demonstrated to be more effective than the best supportive care. The authors observed an antigen-specific immune response and found that it was related to overall survival (Figure 4) [47].

In a T phase 1/2 trial conducted in HNSCC patients, a WT1 peptide-loaded dendritic cell-based vaccine in combination with the OK-432 adjuvant and chemotherapy, was administered. It demonstrated feasibility, safety, and promising clinical efficacy in patients with recurrent or metastatic HNSCC (Figure 4) [48]. CUE-102, an Immuno-STAT, shares remarkable similarities with vaccine-CUE-101. This vaccine is currently being evaluated in clinical trials for various solid malignancies (Figure 4) [5].

### 2.8. Tumor-Associated Antigen Vaccines

In HPV-HNSCC, a vaccine against a mutated epitope of p53 requires custom development, whereas, for the wild-type p53 gene, it could be produced on a large scale. In the phase 1 clinical trial (NCT00404339), the intranodal injection of autologous dendritic cells loaded with wild-type p53 as a tumor peptide-specific p53 vaccine was found to be safe and effective. The two-year disease-free survival rate in a cohort including patients with advanced HNSCC was 88%, and the three-year survival rate was 80%, which outperformed the disease-free survival rate of 70% observed in a similar cohort treated with chemoradiation alone. Although the trial aimed to enroll 50 patients, only 17 were recruited [49]. A phase 1 study (NCT02432963) involving patients with high p53 expression, including one HNSCC, demonstrated the efficacy of p53-expressing modified vaccinia virus Ankara (MVA) (p53MVA) vaccination in combination with pembrolizumab, leading to clinical benefits in select patients. Furthermore, the loss of p53 function can also be targeted for oncolytic therapy using ONYX-15, as discussed earlier [50].

EGFR overexpression is typical of HPV- HNSCC. A vaccine based on dendritic cells containing EGFR fused to a glutathione-S-transferase induced a significant immunity response in mice. A phase 1/2 trial using a recombinant human EGF-rP64K/Montanide ISA 51 vaccine (CIMAvax) and nivolumab for patients with metastatic non-small cell lung cancer or HNSCC is ongoing.

A phase 1 trial (UMIN000000976) showed the safety and advantageous therapeutic potential of survivin-2B peptide vaccination in HLA-A*2402 patients with unresectable, locally advanced, or recurrent oropharyngeal squamous cell carcinoma [51].

### 2.9. Whole Tumor-Based Vaccines

Irradiated NDV-modified autologous tumor cells have been injected intradermally to induce anti-cancer immunity in 20 heterogeneous HNSCC patients 3 months after surgery. The authors reported a 5-year overall survival of 61% and confirmed peripheral immunity after 5 years of disease-free patients [52]. The injection of irradiated autologous tumor cells associated with BCG and vaccine-primed lymph node cells demonstrated efficacy in HNSCC patients [53]. Serial immunological studies demonstrated significant immune responses in vaccinated HNSCC patients with autologous tumors, but it was withdrawn due to not enrolling enough patients [52,53].

Another trial used apoptotic autologous tumor cells fused with dendric cells and administered them to patients with locally advanced HNSCC who had been successfully treated with first-line therapy but were at risk of recurrence or developing a second primary tumor. Serial immunological studies demonstrated measurable immune responses in vaccinated HNSCC patients, specifically targeting the autologous tumor. However, the study has been withdrawn due to impossibility of enrolling enough patients [54].

### 2.10. Tumor Microenvironment Reprogramming

One of the immune escape mechanism of HNSCC is the immunity suppression in the tumor microenvironment. Cancer cells use several immune escape mechanisms, such as PD-1, CTLA-4, IL-6, IL-10, TGF-beta, and STAT-3. The result is the suppression of the CD8+ T Cells and the increase in the Treg and myeloid-derived suppressor cell populations [2,3].

Since the inhibition of myeloid-derived suppressors using a phosphodiesterase-5 (PDE5) inhibitor restores the CD8* cells’ activity, they became a potential target for vaccines [55]. The role of PDE5 inhibitor in potentiating nonspecific and tumor-specific immune responses in HNSCC confirmed by two randomized, double-blinded, placebo-controlled clinical trials (NCT00894413 and NCT00843635), which investigated the use of tadalafil as a PDE5 inhibitor. Another phase 2 trial (NCT01697800) evaluated the combination of tadalafil with conventional therapy in 40 patients with HNSCC between September 2012 and July 2014; however, the results of this trial have not been published. According to ClinicalTrials.gov, among the 25 patients in the tadalafil group, one patient experienced mortality compared to none in the placebo group. A randomized phase 1/2 clinical trial (NCT02544880) was started by the same research group in April 2016, aimed to evaluate the efficacy of tadalafil treatment and Anti-MUC1 in patients with recurrent or second primary HNSCC. Preliminary data reported a safety profile of PDE5 inhibition in HNSCC. This study was motivated by the lack of significant efficacy, as observed in tadalafil monotherapy in previous studies despite the positive enhancement of anti-tumor immune responses [56].

UCPVax is a vaccine against some novel major histocompatibility complexes class II derived from the human telomerase reverse transcriptase that is usually overexpressed in HVP+ HNSCC. This mediation is under study in the VolATIL phase 2 trial (NCT03946358). A similar mechanism of action is used by the vaccine under evaluation in the FOCUS phase 2 trial (NCT05075122) [57].

OX40 is expressed by T cells and enhances their survival and activity, and it can be considered an antagonist of the tumor-suppressive microenvironment. OX40 agonists are under evaluation in HNSCC treatment. Neoadjuvant Anti-OX40 (MEDI6469) demonstrated promising results [57,58]. Another OX40 agonist is under study in a phase 1 trial (NCT04198766 and NCT03739931).

Macrophages are part of the tumor microenvironment, and there are two categories of tumor-associated macrophages, M1 and M2. M1 can kill cancer cells and destroy the extracellular matrix, and M2 has a tumor-promoting action. The transition from M1 to M2 is induced by IL-4, and the opposite switch is induced by IFN-γ [59]. The gamma isoform of phosphoinositide 3-kinase (PI3Kγ) inhibition has been effective in inducing the M1 macrophage expression in animal models [60]. The IPI-549 is a PI3Kγ inhibitor under study as monotherapy or in association with immune checkpoint inhibitor nivolumab for patients with HNSCC in a phase 2 trial (NCT03795610).

Several interleukins, such as IL-15, -2, -7, -12, etc., have been used as targets for HNSCC vaccines development. N-803 (ANKTIVA, ImmunityBio Inc., El Segundo, CA, USA), also known as ATL-803 or Nogapendekin alfa, is an IL-15 agonist bound with its receptor. IT is under evaluation in combination with immune checkpoint inhibitor in a phase 2b study (NCT03228667) with promising preliminary data. Other trials are using N-803 in association with the chimeric antigen receptor T (NCT04847466) or the anti-PD-L1/TGF-beta ‘Trap’ with Bintrafusp alfa (M7824) plus the TriAd vaccine (ETBX-011, ETBX-051, and ETBX-061) (NCT04247282). NKTR-214 (Nektar Therapeutics, San Francisco, CA, USA and Bristol Myers Squibb, New York, NY, USA), also known as Bempegaldesleukin, is an IL-2 pathway stimulator under study in the NCT04936841 phase 2 trial for HNSC. Similarly, ALKS 4230 (Alkermes, Inc., Dublin, Ireland), also known as Nemvaleukin alfa, showed good tolerability in the (NCT04144517) trial [61]. NT-17 is a recombinant ILO-7 called Efineptakin alfa (NeoImmuneTech, NeoImmuneTech, Rockville, MD, USA) under study (NCT04588038). Edodekin alfa, a recombinant Il-12, showed great immunity response in combination with cetuximab in a phase 1/2 trial [62]. The combination of several cytokines in the IRX-2 showed safety and efficacy as a neoadjuvant therapy [63].

TLR stimulation induces natural killer cells activation and antibody cytotoxicity against cancer. Moltolimod, also known as VTX-2337, (APExBio, Houston, TX, USA), is a TRLT8 agonist that increases cetuximab efficacy [64]. Active8 was a multicenter, randomized, double-blinded, placebo-controlled clinical trial comparing the ETREME regimen with placebo or Moltolimod. They observed that adding the vaccine did not significantly improved overall survival and disease-free survival, but a significant benefit was observed in the HPV+ sub-population [65]. EMD 1201081 (Aceragen Inc, Cambridge, MA, USA), also known as HYB-2055, IMO-2055, or IMOxine, is a TLR9 agonist that has been studied in association with cetuximab with no improvement seen in oncological outcomes [66]. Amplivant (AV) (ISA Pharmaceuticals, Leiden, Oegstgeest, The Netherlands) is a TLR-2 agonist that has been conjugated with the HPV E6 to create the HESPeCTA (HPV E Six Peptide Conjugated To Amplivant) vaccine. Its intradermal administration showed safety and efficacy in eliciting immune responses, and further studies are needed the define clinical efficacy [67].

### 2.11. Personalized Cancer Vaccines

Thanks to genome sequencing, it is now possible to analyze the genomic profile of a patient’s cancer and develop a vaccination based on it. YE-NEO-001 (NantBioScience, Inc., Los Angeles, CA, USA) is a recombinant yeast-based vaccine that expresses antigens derived from the patient’s tumor and is under study in a phase 1 trial (NCT03552718). TG4050, an MVA-based therapeutic vaccine based on the myvacTM platform, is under evaluation for locally advanced HNSCC in a phase 1 trial (NCT04183166). AlloVax is a chaperone-rich cell lysate prepared from a patient’s cancer cells associated with AlloStim^TM^ as an adjuvant. This association shows promising results and good tolerability (NCT01998542). MVX-ONCO-1 is made from irradiated autologous tumor cells with a genetically modified cell line called MVX-1 that releases GM-CSF, which exhibited safety and efficacy in HNSCC patients previously treated with nivolumab- or cisplatin-based chemotherapy (NCT02193503). PANDA-VAC is defined as a personalized and adjusted neoantigen peptide vaccine and its association with pembrolizumab is the center of the NCT04266730 phase I clinical trial. VB10.NEO (Nykode Therapeutics ASA, Norway) and NKTR-214, immunotherapy is under evaluation in the NCT03548467 phase 1/2a trial. ATLAS^TM^ is a technology platform for neoantigen selection from tumors. It has been used to make GEN-009, a neoantigen mix made with this technology. It has been administered in association with immune checkpoint inhibitors in a phase 1 trial. It demonstrated good tolerability and promising efficacy [68]. PNeoVCA is a personalized neoantigen peptide-based vaccine under evaluation in association with pembrolizumab (NCT05269381). mRNA-2752 is an mRNA-based vaccine encoding OX40L, IL-23, and IL36γ. It is under evaluation in monotherapy and association with durvalumab (NCT03739931). A different approach uses the in vitro expansion of anti-tumor T-cells extracted from the patient. The phase 2 trial NCT04847466 concerns the association between PD-L1 CAR-NK cells, pembrolizumab, and N-803.

### 2.12. mRNA Vaccines

mRNA can be used to induce the expression of neoantigen peptides and break the immune tolerance to cancer. V941 is a vaccine developed by Moderna and Merck using mRNA-5671, which targets G12D, G12V, G13D, and G12C (the most common KRAS mutations in solid tumors). The NCT03948763 phase 1 trial aims to assess its safety and tolerability either as monotherapy or in combination with pembrolizumab. BNT113 is a HPV16 E7 mRNA and it is currently under study in the phase 1/2 NCT03418480, in combination with HARE-40 (an anti-CD40) and in association with pembrolizumab in the phase 2 NCT04534205 trial. The mRNA used in a vaccine can also encode antibodies. One example of this use with a potential application in head and neck cancer is BNT142, which encodes molecules targeting CD3xCLDN6 (NCT05262530). This vaccine is developed using bispecific T cell engagers called BiTEs, which are bispecific antibodies without the FC region.

The use of mRNA in personalized medicine requires the analysis of tumor antigen expression and the MHC profiling of the patient. Some machine learning algorithms have been used to predict it [9]. Several platforms, such as iNeST (BioNTech SE), have been used to develop BNT121 and BNT122, which are under analysis in solid tumors (NCT02035956, NCT03289962). Moderna has developed the mRNA-4157, which is undergoing testing (NCT03313778).

## 3. Results and Discussion

HPV+ HNSCC usually has a better response to therapy than the HPV- forms [5,69]. The different behavior is due to the carcinogenesis mechanisms induced by the virus or by cigarette smoke and which is easily observable by analyzing gene expression profiles. HPV+ tumors display PIK3CA amplification and CDKN2a or p16 overexpression, they do not overexpress epidermal growth factor receptor (EGFR), and they have the wild type p53. Conversely, non-HPV-related tumors have p53 loss-of-function mutations and EGFR overexpression [69]. A higher percentage of immune cells was also observed in the tumor microenvironment of HPV+ neoplasms [70,71]. The observation that the inflammatory infiltrate is greater in tumors with the best prognosis, i.e., HPV+, correlates with the observations of Zhang et al. in 2021. They observed that HNSCC can be divided into three groups. The first has a greater inflammatory infiltration, less stimulated oncogenic signaling, a greater response to therapy (both chemotherapy and immunotherapy), and, consequently, a better prognosis. The third group consists of those tumors with opposite characteristics, and thus less inflammatory infiltration, more oncogenic mutations, less response to therapy, and worse prognosis [72]. Comparing the characteristics of HPV+ tumors with those of HPV-, regarding the lack of mutation of p53 and EGFR, as well as the greater inflammatory infiltration into HPV+ tumors, it is easy to explain why HPV+ tumors have a better prognosis with medical therapy [69]. Whereby, HPV-positive head and neck cancers have better outcomes compared to HPV-negative diseases. The significant improvement in the application of chemotherapy and radiotherapy protocols has led to good levels of patient treatment and obtaining satisfactory results. [73]. The massive development of vaccines targeting E6 and E7 molecules should consider and compete with those optimal results. Surgery, both traditional and robotic, helps to heal patients in many cases [74], with suggesting the comparison of the results obtained from vaccines with those of the new therapeutic frontiers.

Regarding NPC EBV+ vaccines, clinical efficacy data are limited given the early stage of the trials, yet those medications seem to be well tolerated and able to elicit a selective immune response against the targeted antigens [75]. Obviously, this does not mean that we can declare them to be an effective therapy against HNSCC.

Tumor-associated antigens are not specific to cancer cells but can also be expressed at lower levels in normal tissues. Therefore, vaccines targeting those molecules have low specificity [9].

In addition to evaluating drugs individually, a basic technique for enhancing their efficacy is to combine multiple vaccines. As has already been suggested by Huang et al., we think that future studies should focus on combination therapies with the association of several vaccines or vaccines and other medications, such as the traditional chemotherapy or the new immunotherapy [75]. For example, of the four trials (NCT01256853, NCT01800071, NCT01147991, NCT01094405) that investigated the efficacy of the EBNA1 C-terminal/LMP2 MVA vaccine, only one was concerned with the clinical efficacy of the drug against cancer (NCT01094405). The recruitment was completed, but the results have not been published. Similarly in clinical trial NCT04180215, an improved immune response was observed using the combination of HB-201 and HB-202 vaccines [21].

In our opinion, this observation pushes the future frontiers of research towards the study of the combinations of drugs that prove to be individually effective when creating a therapy that is strong in generating immunogenicity and complete in the antigenic pool. The combination approach also makes sense with the increased action of immunotherapy against immune checkpoints. The actions of drugs such as pembrolizumab are limited by the poor immune responses they generate, despite their actions against a tumor’s immune escape mechanisms. The enhancement of cytotoxic lymphocyte activity through the combination of immune checkpoint inhibitors and therapeutic vaccines is promising, as suggested by the combination of PDS0101 and Pembrolizumab, which has demonstrated such efficacy as to have received Fast Track designation from the FDA in the treatment of HPV16+ in HNSCC [24,25]. Furthermore, we should consider that having an effective drug in monotherapy does not mean that it is more effective than those already on the market. For example, T-VEC was tested in combination with pembrolizumab and the data itself was positive, but the authors who studied the effectiveness of the drug had the foresight to compare the association with the historical data of pembrolizumab alone and showed that there were no significant differences [42]. Consequently, there appears to be no apparent benefit to adding T-VEC to pembrolizumab therapy, and the therapeutic efficacy measured in the trial could be that of pembrolizumab alone. From these data, we can reach two important conclusions: (1) The demonstration of the in vivo efficacy of an immune response against a tumor does not equate to a response in therapeutic terms, and it does not mean that the drug is effective in curing the disease. (2) It is essential to compare these drugs with the current therapeutic gold standard and understand if they are comparable to therapies already in use.

The main goal of vaccine therapy is to induce an immune response against cancer cells. Researchers are still focusing their research on finding vaccines able to activate immunity against tumor-specific antigens. The activation of immune response does not mean therapeutic efficacy. Intuitively, the immune escape mechanisms adopted by cancer cells before vaccine administration could be responsible for tumor cells’ survival after immunity activation against them. For this reason, several researchers are testing anticancer therapeutic vaccines in combination with immune checkpoint escape inhibitors such as Pembrolizumab [24,25,42].

A vaccine to be effective needs to induce an immune response. Adjuvant molecules are extensively used to increase immune activation. Several examples can be used, such as the Candida skin test reagent in the PepCan Vaccine [31,32], Montanide ISA 51 and GM-CSF in LY6K [44,47,58], and AlloStimTM in AlloVax. Their impact on T cell activation may be helpful in increase vaccine efficacy.

Because these drugs act selectively on the immune system and specific antigens, reduced efficacy may occur overall, and it would be important to identify the subpopulation of patients most suitable for receiving the therapeutic vaccine. This principle is the basis of the personalized medicine towards which we are moving. Indeed, the result is that Moltolimod is not effective in the HNSCC population, but the benefit it provides in association with the extreme protocol is significant in HPV+ HNSCC patients [65]. Properly defined personalized vaccines are currently all in experimental stages too early to be able to give a real definition of their clinical efficacy (AlloVax, ATLAS^TM^, PNeoVCA, MVX-ONCO-1, YE-NEO-001, TG4050, VB10.NEO, mRNA-2752, and PANDA-VAC), but certainly, even once their effectiveness has been demonstrated, the widespread diffusion of these therapies is not easy since it is not a matter of distributing a pre-manufactured drug. Furthermore, the question of the diffusion of the technologies necessary for its realization should be mentioned.

Taking, for example, the case of vaccination carried out after surgery with autologous cells: the population taken into consideration is extremely heterogeneous in terms of tumor origin and staging, so it is not easy to evaluate the real clinical efficacy of this practice [52]. Obviously, the list is not limited to this, but even the survivin vaccine, although it showed an immune reaction, did not show therapeutic efficacy. Only one out of ten vaccinated patients showed a partial clinical response, and six out of eight evaluated patients exhibited a noticeable increase in peptide-specific CTLs. However, the investigators noted that the induced CTL response by the vaccine was insufficient to achieve tumor regression [51].

It is premature to make comparisons between standard chemotherapy and vaccine therapy since no phase 3 trials seem to have been completed at the time of writing. For the same reason, vaccine therapy for HNSCC cannot be considered a first-line treatment today. Promising results came from the combination of therapies; for example, the intratumoral administration of ONYX-015 and 5-fluorouracile and cisplatin demonstrated better results than traditional chemotherapy [37]. Those promising results were not confirmed in the phase 3 study that was started in 2001, as no results have been published, even after more than 20 years. The COVID-19 pandemic increased difficulties in conducting clinical trials, making follow-up and experimental protocol application more challenging, sometimes resulting in trial suspensions (NCT03418480).

Future studies must examine not only the conventional efficacy of parenteral administrations but also new administration routes. The NCT04180215 trial is studying, for example, the efficacy of intratumorally administration associated with systemic administration.

## 4. Conclusions

HPV+ HNSCC has better outcomes than HPV. We need to compare the HPV+ HNSCC vaccines with actual results. Data on the clinical efficacy of EBV+ HNSCC are limited given the early stage of the studies. On the other hand, a correct approach to personalized medicine for a population susceptible to the vaccine could produce greater therapeutic advantages.

The actual effectiveness of each new vaccine will have to be compared with the therapeutic successes and health costs of current therapies. Finally, much of data are fragmentary, and numerous studies concerning these vaccines have been aborted, which is a relevant problem in the evaluation of therapeutic vaccines.

## Figures and Tables

**Table 1:** Categories of tumor antigens.

Categories of Tumor Antigens	Description
Classification by Zarour et al.	
Oncofetal	Usually expressed in fetal tissues
Oncoviral	Encoded by virus DNA/RNA
Overexpressed/Accumulated	Expressed in both healthy and neoplastic tissues with higher levels in cancer cells
Cancer-testis	Expressed in adult reproductive tissues physiologically and in neoplastic cells
Linear-restricted	Expressed by specific cancer histotypes
Mutated	Only expressed by cancer
Post translationally altered	Post-transcriptional alteration of molecules
Idiotypic	Highly polymorphic genes expressed in a specific “clonotype” in cancer tissues
Classification by Coulie et al.	
Shared antigens	Expressed both by tumor and healthy cells
Tumor associated antigens	Antigens expressed by tumor and healthy cells that are upregulated in cancers
Tumor specific antigens	Expressed only by tumor cells

**Table 2:** Advantages and disadvantages of vaccine platforms.

Vaccines Platform	Advantages	Disadvantages
Autologous cell-derived	Exposed to all patient tumor antigens Vaccine designed for specific patient disease	Difficult to manufacture. not standardizable. requires sufficient tissue biopsy
Allogenic cell-derived	More potential antigens available; standardization; lower costs	Less personalization
Autologous dendritic cell loaded with tumor antigens	Dendritic cells are the most powerful antigen-presenting cells	Require leukaphereses; require cell culture processing
Peptide vaccines	Easy to produce; easy to store; no viral component	Easy tolerance; rapid degradation in human body; usually require immunogenic adjuvants
DNA vaccines	Use of multiple genes; can be combined with immunostimulatory agents	Modest efficacy; risk of genetic recombination
RNA vaccines	Low levels of side effects; low levels f autoimmune disease	Rapid degradation
Viral vaccines	Induce immune and cell-mediated responses	
